# Does Internet Connect to Social Justice Perception in China?

**DOI:** 10.3389/fpsyg.2022.917039

**Published:** 2022-06-10

**Authors:** Dong Zhou, Jinyu Zhu, Yihan Guo

**Affiliations:** School of Media and Communication, Shanghai Jiao Tong University, Shanghai, China

**Keywords:** internet use, subjective social justice, instrumental variable, regional heterogeneities, China

## Abstract

The world has witnessed an important and dramatic transition during the past decades, with social and economic challenges related to the advancement of digital technologies. Meanwhile, inequalities of distributions of resources and opportunities obstinately exist around the world. This study innovates by utilizing household survey datasets to empirically evaluate the impact of Internet utilization on individual’s perception toward social justice in China. Estimates suggest that Internet utilization generates a significant negative effect on perceived social justice (in general, −5%). In China, there are 1.032 billion Internet users by the end of 2021, accounting for 73% of the total population (China Internet Network Information Center). It suggests that 3.65% of the population, around 5 million people, might consider the current society is injustice if all circumstances remain unchanged. For robustness checks, we not only run multivariate regressions, implemented different model specification, and used alternative measures as well as datasets, but also approached instrumental variable estimation with regional rainfall for causal inference. Consistent conclusions are found. Also, we found stronger negative effects among male, eastern provinces, and urban area samples. To the end, our results shed lights on policy implication, for example, Internet associated public interventions can be focused on justice cultivation and information transparency.

## Introduction

Subjective social justice is one important form of social capitals. Due to its importance, subjective social justice (SSJ, citizens’ perception of social justice) has widely attracted attention of sociologists, economists, psychologists, political scientists, and so on.^1^ A collapse in perceived social justice can erode social capital, undermine stability of society, and hurt social as well as economic development (Sandefur and Laumann, 1998). Better perceptions of justice in organizations can improve the attendance and productivity of employees, reduce turnover, and enhance customer satisfaction and so on (Simons and Roberson, 2003; Schneider et al., 2013). In China, it was reported that 97.2% out of 2,245 respondents agree that the current society lacks social fairness, according to an online survey conducted by *Southern Metropolis Daily.*^2^ The existing literature also found that there is a decline in citizens’ perception of social justice and it has become a concern in China (Chen and Zhang, 2017; Zhang et al., 2018).

Meanwhile, the Internet, as one important new information technology, has progressed dramatically and generated profound impacts on citizens’ lives in China. The number of Internet users has increased from 33.7 million in 2001 to 1.032 billion in 2021. As documented in the existing literature, Internet utilization has affected individuals’ consumption, communication, leisure activities, work, information searching, social connections, and civic involvement (Katz et al., 2001; Wellman et al., 2001; Bargh and McKenna, 2004; Correa et al., 2010; Zhou and Peng, 2017). The psychological effects of Internet use are also studied and proved to be significant, for example, generating loneliness, influencing subjective wellbeing, and lowering political trust (Moy and Scheufele, 2000; Weiser, 2001; Amichai-Hamburger and Ben-Artzi, 2003; Akın, 2012; Zhao and Hu, 2015). However, our understanding of its influences and implications on SSJ is still developing and empirical research on this topic is still rare.

Considering the above two phenomena in China, the decline in perceived social justice, and the expansion of Internet, this study asks a question, “has Internet access, at least partially, invoked the decline in perceived social fairness in China?” and aims to first provide empirical evidence for the relationship between Internet utilization and SSJ. Similar to individual human capital and social backgrounds, information set is also a key determinant in the function of SSJ.^3^ The innovation and generalization of Internet technology have resulted in the world-wide era of information explosion, influenced ways of thinking, shaped one’s information set, and changed one’s perception of the world. Therefore, Internet usage should be most relevant in determining SSJ.

Kinds of media are always the primary sources for individuals to collect information. As the largest developing country, the level of media freedom has been gradually changing in China. Intensely controlled traditional media, relatively open new media, high power distances, and authoritarian government (Li, 2004; Kennedy, 2009; King et al., 2013) construct its special media and social environments. Internet with relatively less information censored and faster information communication must differ from the traditional media in affecting perceptions of social justice. Nevertheless, inconsistent information from Internet against positive propaganda on traditional media could generate uncertainties in one’s information set, so that the SSJ is influenced. Lee (2017) found that there is a U-shaped relationship between media freedom and social capital and exists a critical threshold level across which public voice and media will contribute to better social capital. Some countries are located at the left of the threshold, where media plays a negative role in improving social capital. Finding out how Internet utilization correlates with SSJ can help locate China on the U-curve and contribute to better policy implications. In this study, we employed the 2015 and 2010 waves of Chinese General Social Survey (CGSS) to first evaluate the impacts of Internet utilization on individuals’ perception of social justice and innovate by implementing the instrumental variable approach for causal inference.

## Theoretical Background

The discussion of social justice can be traced back to *Utopia* by Plato, in which Plato states that social justice can be achieved when all social classes in a society are in a harmonious relationship. In other words, social justice should be a proper and reasonable state, not only with equitable distribution of social resources and rights, but also with impartial relationship among social members. Rawls (1971) stated that social justice not only ensures that everyone has similar rights to equality and freedom, but also makes sure that the powerful people have the obligation to give the disadvantage of a variety of basic compensations so that the weak people can have opportunities to participate in social competition like the strong ones. Perceived social justice, in this way, is a kind of psychological feeling which arises from one’s judgment of social justice, and it is based on one’s view of the relationship between the “should be” and “the state” of social justice.

The existing literature has continuously documented important consequences of perceived social justice in many domains of social life in the past decades. For example, positive perceptions of social justice, in particular procedural justice and distributive justice, are important matters for legitimacy, law implementation, and politics, because the positive SSJ contributes to better public cooperation and political support (Tyler et al., 1985; Lind et al., 1993; Tyler, 2003). Also, SSJ plays significant roles in economic development, i.e., managing organizations (Cropanzano and Greenberg, 1997; Cohen-Charash and Spector, 2001; Aryee et al., 2002; Fang et al., 2011; Robbins et al., 2012) and facilitating economic transactions (Kahneman et al., 1986; Fehr and Schmidt, 1999). In addition, positive feelings of social justice can cultivate intimate relationships and construct better social networks (Mikula and Lerner, 1994; Lerner, 2003; Schmitt et al., 2005). Overall, most of the existing studies focus on the effects of social justice and reactions to injustice, but studies on determinants of SSJ are relatively limited.

Different individuals with different cognitive abilities, human capital, standpoints, and social backgrounds are likely to form different judgments about social justice. In general, there are two intertwined interpretations for the formation of SSJ, namely, rationalism and intuitionalism. First, rationalism emphasizes that during cognitive processes, judgments of events are based on careful evaluation of available relevant information (Van den Bos et al., 2001). People’s perceived social equity depends on, comparing outcome/input ratios with others, references to evaluate whether they are treated fairly (Adams, 1963; Folger, 1986). Perceptions of distributive fairness, procedural fairness, and opportunity fairness are influenced by information available and one’s cognitive ability. Depending on better cognitive ability, one can accurately process information received, make comparisons, and then form reasonable judgments. Second, the intuitionalism suggests that in some situations, people’s impulsive reactions lead to quick perceptions of social justice. Once these quick judgments are made, they would be stored and generate path dependences, which means that perceived fairness will be difficult to change once it is formed (Lind et al., 1998). Most of the social psychologists believe that even for the deliberate decision-making process, citizens’ judgment process hardly maintains being a pure rational system but switches to be an impulsive and quick automatic approach (Schwarz and Clore, 1996; Van den Bos, 2003; De Cremer et al., 2008). Individuals, under uncertainty, will turn to other fragmented information or the rule of thumb (Van den Bos et al., 1997; Van den Bos, 2003), i.e., changing from thoughtful thinking to relying on temporary information and affective responses.

As discussed above, the essence of SSJ is still subjective perceptions of outcomes as well as opportunities across persons, and judgments about the justifications of using one procedure rather than another. Just as human capital and individual background, information set is also a key determinant in the function of SSJ, no matter for a rational agent or an impulsive citizen. In all, SSJ is a function of information and factors depending on how citizens process information, for example, personal human capital, cognitive ability, family backgrounds, social status, civic engagement level, and macro-environments.

Attributable to information technology innovation and Internet generalization, this is an era of information explosion. Individual’s information set has been dramatically enlarged through Internet utilization. At present, people become producers who both use or consume information from Internet and also produce or create information for others to use online (Coleman et al., 2009; Bird, 2011). Internet coverage has tightened the world, strengthened users’ social connectedness, and thus mobilized civil engagement (Parks and Floyd, 1996; Rheingold, 2000). Information, i.e., news or knowledge, can be searched online and easily obtained. From this perspective, Internet allows the world to become more transparent and reduces the information uncertainties (Weiser, 2001; Bargh and McKenna, 2004; Alcaraz-Quiles et al., 2014). Even individuals with lower cognitive ability can make judgments with rich information if they can access to Internet and collect information.

However, there is another possibility. Castells (1996) stated that the Internet technology has diversified information sources, decentralized information usage or reception, and fragmented information. Plausibly, the society evolves into a segmented one in which people have a greater chance to encounter incomplete or pruned news. Instead of getting close to the truth beyond manipulated reports, citizens are overwhelmed by the kaleidoscopic stories online. It turns to be more difficult for one to differentiate. Under such situations, individuals are likely to form their perceptions of social justice based on affection and experience. Both rationalism and intuitionalism can work. Therefore, Internet usage must have played a role in perceived social fairness. Its role might be different in different stages of economic and democratic development and should be different for individuals with different cognitive ability and screening ability.

In the scenario of China, the biggest developing country, network information technology has significantly benefited the economic development for the past 20 years and almost influenced all perspectives of social life of Chinese residents. On the one hand, the economy has experienced sustainably rapid growth and citizens’ standard living has been largely enhanced. There are great strides in public facilities and services because of benevolent government (Liu et al., 2010). Political institution and governmental governance have also become more efficient. Therefore, positive motions toward social equity can be promoted, thereby improving social life. On the other hand, income distribution and redistribution become concerns. Problems, such as unbalanced regional development, rural-urban income inequality, and opportunity inequality, turn social justice to be the focus (Li et al., 2013; Xie and Zhou, 2014). Owing to its advantage of simultaneous and efficient information dissemination, Internet has lowered the information searching cost and has enlarged the available information set for citizens. News on income inequality and social stratification are widely reported. Also, better society will produce “critical citizens” holding higher requirements and expectations (Norris, 2011).

Meanwhile, Munchhausen through Internet becomes a common phenomenon (Lawlor and Kirakowski, 2017). This is also the case of China. Overexaggerated information on wealth and power abused by authorities or minorities floods the Internet. Wealth or status comparisons can downplay one’s perception of social equity. It can generate psychological deficits in the public. Such psychological deficits include a poor sense of self, unmet needs, and personal trauma. Those deficits could even trigger hatred in irrational scenarios since Internet allows not only more transparence but also exaggerated stories. Social judgment of justice, correspondingly, can be affected passively. Also, sympathy toward the disadvantages or unfairly treated groups arouses empathy and negative judgment on social fairness. Nevertheless, the popularity of online news about conflicts, unjust treatment, or unverified stories could create an air of suspicion among the entire incumbent officers and law-enforcing departments. All these produce an image of procedural unfairness and interactional injustice where citizens have greater probability of encountering unfair treatments.

Furthermore, China has its own unique media environment. The new media and traditional media are divided and they sometimes provide inconsistent information. Although, currently, information searching and receiving become more efficient and faster, perfect transparency is still hard to achieve in China. Traditional media is majorly led by the incumbent government and has exclusively focused on the positive propaganda (Li, 2004; Kennedy, 2009). Chen and Shi (2001) argued that positive propaganda would work if media message recipients accept information conveyed blindly without any reasoning, but the intent and practice to saturate people with message bombardment force people to think a great deal. The continued propaganda bombardment and the intent silence of the official media on political scandals, corruptive behaviors of public officials, and social injustice events also drive people to seek alternative information sources. Internet is a relatively more efficient source, because even it suffers censorship, it is not as strong as traditional media (King et al., 2013). Chinese citizens increasingly use online social media to express views and spread “unofficial” news while giving more credibility to online rumors (Saich, 2012). When there are inconsistencies between the formal news reports and online versions, cognitive dissonances will be generated and judgments of social justice are made with the rules of thumb. In other words, given such an uncertain circumstance, citizens are more likely to turn to impulsive reactions and judge the social fairness with the rule of thumb.

Therefore, it is an empirical question to evaluate the above two opposite directions which plays a dominant role. As discussed above, it is likely that the negative impact might play a dominant role and there should be heterogeneous effects of Internet use on SSJ among different subgroups of population as well as among different media sources.

## Data, Measures, and Statistics

Our main data are drawn from the 2015 and 2010 waves of CGSS, because it is a national representative with randomized large sample size and it allows us to make implications and general validations for the nation which cannot be easily achieved by small sample. The existing literature has utilized the CGSS to study the impacts of Internet use on political trust, social value, social identity, and rural development (Wang and Wu, 2014; Su and Huang, 2015), but not the relationship between Internet utilization and SSJ.

This survey is administered by the Data and Research Center of Renmin University and systematically tracks changes in the nationwide social structure and quality of life in China. Importantly, it provides detailed demographic, economic, and social information. The most pertinent to our study is that respondents report their media use, ways of information collection, and self-perceived social justice. These variables allow us to answer our research question empirically. Our empirical sample covers 7953 respondents aged from 17 to 65 years from 129 countries over 27 provinces. In addition, we collected numbers of broadband access ports at the provincial level in 2015 from official statistic yearbooks, collected provincial rainfall information, and merged them with the individual data. These two variables are used for instrumental variable estimation.

### Dependent Variables

Respondents were asked to answer “How do you think about the current social justice in China” and choose a value between 1 and 5 ranging from low social justice perception to high social justice perception, where 5 means “firmly agree this is a fair society,” 4 means “agree that the society is relatively fair,” 3 means “neutral,” 2 means “our society is not so fair,” and 1 means “our society is totally not fair at all,” respectively. This survey question is to obtain a general evaluation of social justice. Based on its answer format, we constructed two variables for measuring SSJ. One is a qualitative indicator, in which value 1 represents the respondent agrees we have a fair society (the answer is 4 and 5) and 0 otherwise. The other is a categorical variable that maintains the categorical nature of the above answers. Definitions of all the variables are shown in Table A1. Table 1 presents both statistics. Statistics display that most of the Chinese residents do not agree that the current society is just in 2015, i.e., approximately 52.87%. As a result, 22.76% of them think that the current society is neither just nor not just and 47.13% of the surveyed population support that we have justice in the current society. For the dummy measure, we run linear probability regressions and use Ordered Probit estimations for the scale measure. Empirically, both ways provide qualitatively consistent findings.

**TABLE 1:** Summary of main descriptive statistics.

Variables	Obs.	Mean	*SD*	Min	Max
Subjective social justice (1,0)	7,953	0.471	0.499	0	1
Subjective social justice (1–5)					
*Complete injustice; = 1*	7,953	0.062	0.242	0	1
*Not very justice; = 2*	7,953	0.239	0.426	0	1
*Neither justice nor injustice; = 3*	7,953	0.228	0.419	0	1
*Relative justice; = 4*	7,953	0.444	0.497	0	1
*Complete justice; = 5*	7,953	0.027	0.162	0	1
Internet utilization (Yes = 1; No = 0)	7,953	0.553	0.497	0	1
**Internet utilization frequency**					
*Never use; = 1*	7,953	0.447	0.497	0	1
*Barely use; = 2*	7,953	0.080	0.271	0	1
*Sometimes use; = 3*	7,953	0.084	0.277	0	1
*Often use; = 4*	7,953	0.167	0.373	0	1
*Very often; = 5*	7,953	0.222	0.417	0	1
Province level: Number of	7,953	19.96	13.14	0.51	47.66
Broadband access ports (Million)					
Self-evaluated social status (1–5)					
Far below the average = 1	7,953	0.145	0.352	0	1
Below the average = 2	7,953	0.324	0.468	0	1
The average = 3	7,953	0.467	0.499	0	1
Higher than the average = 4	7,953	0.058	0.234	0	1
Far higher than the average = 5	7,953	0.006	0.078	0	1
**Healthy status**					
*Very unhealthy; = 1*	7,953	0.023	0.149	0	1
*Unhealthy; = 2*	7,953	0.120	0.325	0	1
*So-so; = 3*	7,953	0.204	0.403	0	1
*Healthy; = 4*	7,953	0.402	0.490	0	1
*Very healthy; = 5*	7,953	0.251	0.434	0	1
**Education attainment**					
*Illiterate; = 1*	7,953	0.091	0.288	0	1
*Primary education; = 2*	7,953	0.210	0.407	0	1
*Junior education; = 3*	7,953	0.314	0.464	0	1
*Senior education; = 4*	7,953	0.201	0.401	0	1
*Higher education; = 5*	7,953	0.184	0.388	0	1
Female	7,953	0.528	0.499	0	1
Han ethnicity	7,953	0.921	0.270	0	1
Age	7,953	44.630	12.952	18	65

### Key Independent Variables

In the survey, respondents were asked about the information on their Internet usage. The first survey question we used is that “how often you use Internet (including mobile Internet)?” The answer is also reported with a value between 1 and 5 representing frequencies of usage from low to high, where 1 means “never use,” 2 means “barely use,” 3 means “sometimes use,” 4 means “often use,” and 5 means “very often use.” Like the SSJ, we constructed two measures for Internet usage. One is a scale variable (we named the variable, *Internet Utilization Frequency*) considering values between 1 and 5 and the other is a dummy variable (we named it *Internet Utilization*) that takes value 1 if the respondent had used the Internet during the previous year and 0 otherwise. A total of 55.27% of the respondents reported previous Internet use (see Table 1).

**FIGURE 1:**
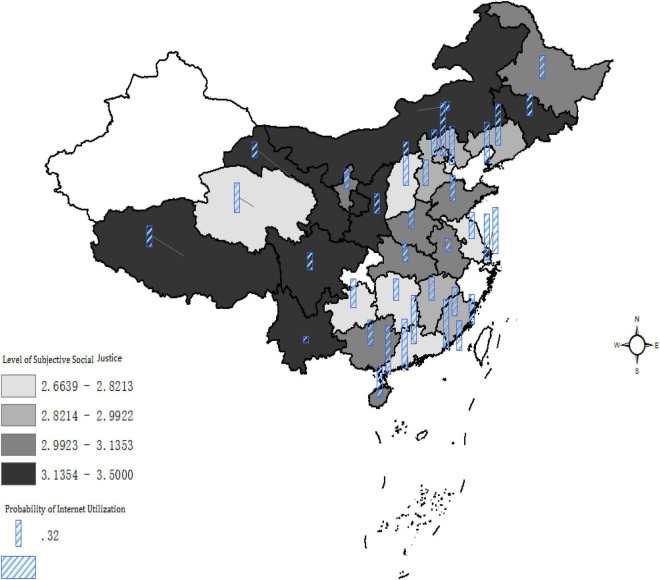
The negative association between Internet use and subjective social justice. The graph is computed based on authors’ calculation.

Figure 1 visually illustrates geographical variations of the average value of perception of social justice and Internet utilization at the provincial level. The blue bars indicate the Internet utilization rate for each province. The higher the bar is, the larger the Internet coverage is. Color in the lower layer areas shows the provincial average level of SSJ. The dark the color is, the higher the level is. In general, the province with a higher bar is with light color. It suggests that the provinces with more people using Internet are more likely to exhibit a lower level of SSJ. A negative correlation seems to be visually evident.

The second survey question we used is that “which channel (media) is the primary channel you use for information collection.” As a result, 32.85% of respondents identified the Internet (including mobile Internet) as their main information source, 62.92% of them still selected television and radio as their primary information source, and 2.27% of observations report that newspaper is their main information source. As discussed above, sources of information collection matter and Internet utilization are expected to generate a more significantly negative influence on SSJ.

### Other Control Variables

First, control variables included gender, age, ethnicity, natural log of personal income, comparison income measured by the difference between one’s own household earning and the local mean, self-reported health condition measured by a scale variable ranging from 1 to 5 from very unhealthy to very healthy, educational attainment, marriage status, the urban dummy if one lives in urban area then the value is 1, and a group of province dummies controlling regional fixed effects. These basic socio-economic and demographic variables shape one’s cognitive ability, personality, value, and attitude, and they should explain part of the variance of subjective social fairness.

Second, control variables that reflect ones’ status of social identity and civic engagement are considered. They include religion situation (whether a person is an atheist), occupation characteristic (whether one currently holds a government position), one’s self-evaluated economic social status (a scale variable from low to high social status valuing 1 to 5), one’s level of sociability (it is self-assessed and is a scale variable from low to high sociability valuing 1 to 5), and one’s political party involvement (it is measured by the party membership, including communists, other party memberships, or non-party memberships). We only provided statistical description for the main variables in Table 2 and more data are available upon request.

**TABLE 2:** Pearson correlation coefficients’ matrix.

Pearson correlation coefficients	SSJ (Dummy)	SSJ (Scale)	Internet utilization	Internet utilization frequency	Urban dummy	Male dummy
Subjective social justice (Dummy)	1					
Subjective social justice (Scale)	0.8601***	1				
Internet utilization Frequency	–0.0708***	–0.0679***	1			
Internet utilization	–0.0705***	–0.0678***	0.8814***	1		
Urban dummy	–0.0384***	–0.0343***	0.3256***	0.3072***	1	
Male dummy	0.0304***	0.0238	0.0577***	0.0587***	0.0198	1

***Indicates the significance level at 1%.

In all our empirical models, we controlled all the above variables. After partialling out the effects of the above general social variables on individuals’ perception of social justice, consistently significant coefficients of interest will support the existence of effects of Internet utilization on SSJ. For robustness checking, we have used alternative data, and employed various combinations of controls for model specifications.

## Empirical Analytic Strategies and Estimation Results

### Pearson Correlation Coefficient Matrix

Pearson correlations among dependent variables and interested independent variables are presented in Table 2. An inspection of the correlations between the frequency of Internet usage variables and subjective social fairness revealed that they are negatively related. We also considered the urban-rural disparity and gender gap. The urban dummy is negatively correlated with perceptions of social fairness, while it is positively correlated with Internet usage. In general, Internet coverage is more general in urban areas and urban citizens use Internet more frequently. On average, the level of subjective agreeing social justice is lower in urban areas. Also, males use Internet more than females and exhibit a relatively higher SSJ level before considering other socioeconomic characteristics.

### The Effect of Internet Utilization on Subjective Social Justice

We run multivariate OLS and Ordered Probit estimations to evaluate the effects of Internet usage on individuals’ perception of social justice. Estimates of interested variables are presented in Table 3. The general form of empirical models can be formalized as follows:

**TABLE 3:** The effect of Internet utilization on subjective social justice.

	Subjective social justice (dummy 0–1)		Subjective social justice	
			(Ordinary 1–5 Scale)	
		
	OLS	Probit	OLS	Ordered probit
Internet utilization	–0.050***	–0.132***	–0.118***	–0.136***
	(0.016)	(0.041)	(0.031)	(0.035)
Comparative income	0.0000 000407**	0.0000 00121*	0.0000 000698	0.0000 000991
	(0.00000 00203)	(0.0000 000735)	(0.0000 000462)	(0.00000 00617)
Annual income	0.002	0.006	0.005*	0.006*
	(0.002)	(0.004)	(0.003)	(0.003)
**Educational attainment base group: Ilterate**				
Primary school	–0.024	–0.063	–0.044	–0.055
	(0.022)	(0.058)	(0.045)	(0.051)
Junior high	–0.029	–0.076	–0.053	–0.063
	(0.023)	(0.059)	(0.046)	(0.052)
Senior high	–0.028	–0.077	–0.037	–0.051
	(0.025)	(0.067)	(0.051)	(0.057)
College and above	0.002	0.002	0.060	0.047
	(0.029)	(0.077)	(0.058)	(0.065)
**Socioeconomic status (SES) base group: Lower class**				
Lower middle class	0.081***	0.219***	0.248***	0.259***
	(0.017)	(0.047)	(0.038)	(0.041)
Middle class.	0.167***	0.443***	0.426***	0.460***
	(0.017)	(0.046)	(0.037)	(0.041)
Upper middle	0.222***	0.588***	0.539***	0.595***
	(0.028)	(0.075)	(0.058)	(0.066)
Upper class.	0.192***	0.514***	0.494***	0.616***
	(0.073)	(0.191)	(0.172)	(0.211)
No religion	0.025	0.067	0.038	0.034
	(0.018)	(0.049)	(0.038)	(0.042)
Male	0.027**	0.070**	0.045**	0.057**
	(0.012)	(0.031)	(0.023)	(0.026)
Urban	–0.025*	–0.065*	–0.072**	–0.073**
	(0.014)	(0.037)	(0.028)	(0.031)
Observations	7,953	7,953	7,953	7,953
R-squared	0.060		0.075	

Robust standard errors in parentheses; ***p < 0.01, **p < 0.05, *p < 0.1; For all estimations, other controls include age, ethnicity, party membership, marital status, health status, household wealth, sociability level, government position, provincial fixed effects and provincial level variables.


(1) $$\mathrm{Subjecitve}\,\mathrm{Social}\,{\mathrm{Justice}}_{i}=f\,\left(I\,n\,t\,e\,r\,n\,e\,t\,U\,s\,e_{i},X_{i}\right)+\epsilon_{i}$$

where *X* is the vector of control variables including individual socio-economic and demographic characteristics, civic engagement associated variables, personal characteristics mentioned above (e.g., gender dummy, ethnicity, age, health condition, educational attainment, earnings, and marriage status), and provincial fixed effect. ε is the disturbance term. “*Subjective Social Justice*” is measured by the qualitative indicator and the categorical variable, considering a value between 1 and 5 measuring levels. We estimated Ordered Probit regressions for scale measure and linear probability regressions for the dummy measure. It is noted that OLS and Ordered Probit estimations are both implemented for all the possible combinations of dependent and independent variables and all estimates are qualitatively identical.

We adopted the constructed dummy variable indicating previous Internet use experience, Internet Utilization, and the qualitative indicator for whether one agrees whether the society is just or not. The results are presented in Table 3. Each column represents one specific estimation result. Only estimated parameters for our interested control variables are presented. Full results are available upon request. According to estimates presented in Table 3, Internet users are more likely to think that current Chinese society has a lack of justice than individuals without Internet access as the negative coefficient for Internet utilization is statistically significant (–5%). The partially out effects of socio-economic and demographic characteristics, the key coefficients imply that Internet use can generate a negative impact on respondents’ perception of social justice. It has initially demonstrated the point in the relevant literature that Internet use affects a person’s sense of fairness. During the current transition of economy and social development, China, as one of the developing countries, has experienced unbalanced regional development, income inequality, and opportunity inequality. Benefits of the reform and opening-up have not been equally distributed. As mentioned earlier, the use of the Internet can affect socioeconomic factors such as personal consumption, work level, and citizen participation. Nevertheless, the explosion of information through Internet can also affect the modes of thought of different people. Social media online often overly exaggerates negative news, in the context of Internet; ordinary news are more likely to reach people of higher-class, which will affect their perception of social equity. As a result, after the connection of Internet, residents start to have more information and comparison generates dissatisfaction and perceptions against social fairness. It may affect the psychology, influence social supports of citizens, and then impact social stability.

Second, as presented in Table 2, we showed that the urban and female residents, on average, exhibit lower levels of perceptions of social fairness, and this is also confirmed in the regressions. As documented, the rural-urban bifurcations in income and social welfare are significant (Wan et al., 2006). Some studies reveal similar results that rural respondents are significantly more likely to consider the Chinese health care system equitable, which is also a manifestation of SSJ (Lv et al., 2020). Social lives are different between rural and urban areas. Urban residents are more likely to use Internet products and receive information, and technology continues to improve while broadband access is still a problem in a wide range of rural areas. In other words, there is a digital division between rural and urban China persists. It is possible that the mechanisms of the impact of Internet utilization on perceptions differ among rural and urban areas. Educational attainment is uncorrelated with respondents’ SSJ. In Chinese society, social status is largely related to household wealth and whether one is an influential official. The estimates show that when the respondent thinks that he or she belongs to a higher social level, he or she agrees that the social justice level is higher. Vested interest theory also explains the same fact. Finally, after controlling self-evaluated household social status, individuals’ income and comparative income become less significant.

Third, potential unobserved factors may cause deviations and affect the estimation results. To deal with endogenous issues, we then resorted to the instrumental variable approach for a causality check. This requires an instrument that is correlated with the interested independent variable Internet use but not directly related to the dependent variable. We adopted the regional rainfall and provincial Internet coverage rate as the instrument, respectively. Excessive rainfall will damage local communication devices, which will hinder their Internet use. The higher the provincial Internet coverage, the better the local network facilities and the higher the probability that residents will use the network. The instruments are to some extent correlated with one’s intensity of using Internet but are not straightly correlated with one’s current perception toward social justice. The estimations can be formalized using a two-stage least square procedure as follows:


(2) $$I\,n\,t\,e\,r\,n\,e\,t\,U\,t\,i\,l\,i\,z\,a\,t\,i\,o\,n_{i}=\alpha_{i}+\beta \cdot I\,V_{i}+\gamma \cdot X+\varepsilon_{i},$$

Subjecitve Social Justice_*i*_


(3) $$=\lambda +\delta \cdot P\,r\,e\,d\,i\,c\,t\,e\,d\,I\,n\,t\,e\,r\,n\,e\,t\,U\,t\,i\,l\,i\,z\,a\,t\,i\,o\,n_{i}+\kappa \cdot X+\varepsilon_{i}$$

The estimation results are presented in Table 4. SSJ variables and Internet use are measured with dummies and we applied the linear probability model with the instrument. While individuals living in a region with a high coverage rate are more likely to use Internet or mobile Internet during casual time, individuals living in a region with extraordinary rainfall are less likely to access Internet. The results of the first phase show that if the regional rainfall increases by 10 billion cubic meters, the probability of local people going online will decrease by 0.07%, and the network coverage variable presents similar results. For both instruments, the first stage estimation is strongly significant and the F-statistics (weak instrument test) is larger than 10, although the sizes are not large. Moreover, four estimates consistently support the robustness of our conclusion that Internet use negatively impacts SSJ in China. Our analysis above has confirmed that Internet use has partially made SSJ to fall in China.

**TABLE 4:** Instrumental variable approach estimates.

Dummy measure	First stage	IV estimate
Provincial internet Coverage rate as IV	0.00185*** (0.00053)	–7.25900*** (2.72937)
*F*-test	12.2829	
Provincial rainfall (Billion Cu.M) as IV	–0.00090*** (0.00012)	–6.45053*** (2.42538)
*F*-test	13.6631	
Observations	7,990	7,960

Robust standard errors in parentheses. ***p < 0.01; 2. All regression controls for all variates as regression of Table 3, the province dummies are excluded since the instruments are measured at provincial level and variations will be smoothened out by the provincial fixed effect controlling.

### Robustness Checks

In Table 5, we run estimations with different subsamples and presented heterogeneous effects. The third row shows average levels of social justice perceptions across different groups. In general, the more developed areas (e.g., the urban and eastern areas), the lower the level is. Each column indicates one specific regression. Consistently, we observed a significant negative effect of Internet usage on individual’s SSJ with two exceptions, the rural and central provinces’ samples. However, when we further utilized the 2010 wave of CGSS and re-estimate the effect of Internet usage on SSJ as one way to check robustness, the negative effects are significant for the rural residents and central provinces’ residents. Please see Table 6 for pieces of evidence. We have tested different measures for Internet usage and run OLS and Ordered Probit regressions, respectively. For all the estimates, the interested estimates are significantly negative. Consistent findings are found. Overall, the negative correlation between Internet use and SSJ is significant. Instrumental variable estimates imply that the negative effect is causal. More results are available upon request.

**TABLE 5:** Heterogeneous effects within different subsamples.

Dependent variable:		Subjective social justice (dummy measure)							
Sample	All		Males	Females	Urban	Rural	Eastern	Central	Western
*The means of dependent variables*	*0.466*		*0.482*	*0.452*	*0.444*	*0.483*	*0.427*	*0.483*	*0.519*
Regression	(1)		(2)	(3)	(4)	(5)	(6)	(7)	(8)
Internet utilization	–0.050***		–0.067***	–0.036*	–0.076***	–0.026	–0.087***	0.003	–0.072**
	(0.016)		(0.022)	(0.022)	(0.025)	(0.020)	(0.024)	(0.026)	(0.033)
Constant	–1.594***		–1.333*	–1.554**	0.161	–2.640***	4.181***	–3.674***	–0.068
	(0.544)		(0.789)	(0.756)	(1.083)	(0.806)	(0.887)	(1.059)	(0.809)
Observations	7,953		3,752	4,201	3,392	4,561	3,396	2,932	1,625
R-squared	0.060		0.062	0.068	0.059	0.077	0.069	0.053	0.073

Robust standard errors in parentheses***p < 0.01, **p < 0.05, *p < 0.1. Only interested estimates are presented; All regression controls for all variates as regression of Table 3. The division of the eastern, central, and western regions of China is based on official data from the National Bureau of Statistics of China.

**TABLE 6:** Robustness check with 2010 CGSS.

Sample	All	Urban	Rural

Panel A (OLS estimates)	Subjective social justice (dummy 0–1)		
Internet utilization	–0.061***	–0.050***	–0.101***
	(0.015)	(0.017)	(0.029)
Constant	0.393***	0.232**	0.731***
	(0.098)	(0.106)	(0.213)
Observations	8,126	4,898	3,228
R-squared	0.062	0.062	0.058
Panel B (OLS estimates)	**Subjective social justice (dummy 0–1)**		
**Internet utilization frequency base group: Never use**			
*Barely use*	–0.057***	–0.034	–0.128***
	(0.021)	(0.024)	(0.042)
*Sometimes*	–0.055***	–0.053**	–0.062
	(0.021)	(0.024)	(0.047)
*Often*	–0.052**	–0.039*	–0.118**
	(0.020)	(0.023)	(0.051)
*Very often*	–0.090***	–0.088***	–0.084
	(0.022)	(0.024)	(0.062)
Constant	0.401***	0.239**	0.731***
	(0.098)	(0.106)	(0.214)
Observations	8,126	4,898	3,228
R-squared	0.062	0.064	0.058
**OLS estimates when treat *internet utilization frequency* as a continuous variable**			
Internet utilization frequency	–0.021***	–0.019***	–0.032***
	(0.005)	(0.006)	(0.041)
Observations	8,126	4,898	3,228
R-squared	0.062	0.063	0.057
**Panel C (ordered probit estimates)**	**Subjective social justice (Ordinary 1–5 scale)**		
**Sample**	**All**	**Urban**	**Rural**
Internet utilization	–0.101***	–0.064	–0.241***
	(0.032)	(0.039)	(0.063)
Observations	8,114	4,895	3,219
Pseudo R-squared	0.0264	0.0274	0.0229

Robust standard errors in parentheses; ***p < 0.01, **p < 0.05, *p < 0.1; For all estimations, other controls include ethnicity, gender, education attainment, natural log of personal income, comparison income, risk aversion level, marital status, health status, religion, subjective social status, party membership, occupation, and provincial fixed effects. More results are upon required.

## Concluding Remarks and Outlook

As described above, information and communication technology have generated crucial influences on economic and social development around the world. About 4.66 billion people around the world used the Internet in January 2021 and global Internet penetration stands at 59.5% (Kemp, 2021). To understand the potentials for Internet, technologies to affect social behaviors will be beneficial for policy makers and practitioners. As China’s marketization and digitalization processes have improved, changes have taken place. Its influencing factors include traditional social strata and distribution of benefits. Also, Internet has increasingly become a trusted and sought source of information and has brought profound influences on current societies. Among the extensive literature exploring the influences of Internet use, there has been a relatively small amount of research examining its impacts on SSJ and empirical evaluation is rare.

In our study, we first explored the role played by Internet use on perceived social fairness in China empirically. We also pioneeringly addressed endogeneity problems through using numbers of provincial broadband access ports and rainfall levels as instrumental variables to run two-stage least square estimation. Estimates consistently suggest that there exist significant negative effects of Internet use on SSJ and the marginal effect is stronger in relatively developed regions. On the margins, Internet utilization will lower individual’s perceived social fairness by 5% controlling ethnicity, gender, education attainment, income, comparison income, marital status, health status, religion, subjective social status, party membership, occupation, and provincial fixed effects. In 2015, there are 0.67 billion Internet users and the estimate implies that Internet connection can affect the SSJ of 3.34 million people. There are 1.032 billion Internet users by the end of 2021, accounting for 73% of the total population. It suggests that 3.65% of the population, around 5 million people, might consider the current society as injustice if all circumstances remain unchanged. Moreover, considering the worldwide digitalization trend and inequality of resources or opportunities, our results also highlight the importance and direction of public Internet intervention across countries.

**FIGURE 2:**
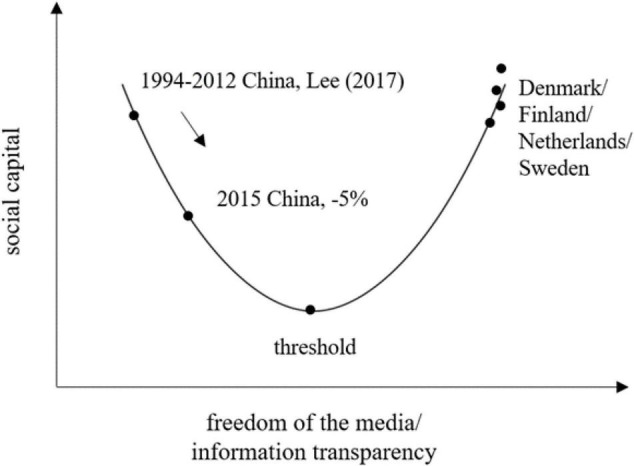
The U-relationship between media freedom and social capital. The graph is computed based on authors’ calculation and Lee (2017).

The negative effect we found does not imply that the government should strengthen the media controlling or block people from Internet accessing. It is probably the opposite. Lee (2017) used cross-country panel data and found that there is a U-shaped relationship between media freedom and social trust. Some countries are located at the right of the threshold, where media plays a positive role in improving social capital. However, according to our empirical evidence, China is currently located at the left side of the threshold (see Figure 2). Speech suppression and uncertainties caused by inconsistent reports hinder the affirmative roles of media usage in social capital. Hence, further enhancement of media freedom and allowance of more public transparency are needed to step across the threshold and cultivate the affirmative roles of new media usage in social capital.

Finally, we would like to mention several pitfalls of this study. One is that, although the robustness checks suggest the validity of the effects, we cannot rule out all the possibilities that there are potentially endogenous concerns, for example, people who score the social justice lower are more likely use Internet frequently for information searching. Future research can be carried out with panel datasets for causality. Another pitfall is that we only focused on establishing the negative effect of Internet usage on SSJ controlling potential contaminating factors as many as possible. Our future research will investigate more specific channels aside from information collection in the mechanism through which individual Internet use matters when more datasets become available. In addition, our dependent variable, Subjective Social Justice, is measured using a single survey item. This approach is common in the literature and is supported by recent studies (Sun et al., 2013; Hu et al., 2015; Zhu et al., 2020; Yang et al., 2021). However, future research should consider using multiple survey items to capture Chinese people’s perception toward social justice from different conceptual aspects, such as distribution and judicial justice.

## Data Availability Statement

Publicly available datasets were analyzed in this study. This data can be found here: http://cgss.ruc.edu.cn/index.htm.

## Author Contributions

DZ was in charge of writing and data analysis. JZ was in charge of theoretical background and review. YG was in charge of data cleaning and running regressions. All authors contributed to the article and approved the submitted version.

## Conflict of Interest

The authors declare that the research was conducted in the absence of any commercial or financial relationships that could be construed as a potential conflict of interest.

## Publisher’s Note

All claims expressed in this article are solely those of the authors and do not necessarily represent those of their affiliated organizations, or those of the publisher, the editors and the reviewers. Any product that may be evaluated in this article, or claim that may be made by its manufacturer, is not guaranteed or endorsed by the publisher.

## Footnotes

1 Subjective social justice states an overall perception toward diverse states of the society, e.g., income distribution, judicial justice, class privileges, opportunities and so on. It is a kind of psychological feeling and based on one’s view of the relationship between the “should be” and “the state” of social justice. For example, if people feel that their input/output ratio are not the same as other people, a sense of injustice or unfairness will be generated. Subjective social justice is worded as the way as subjective wellbeing which refers to personal perceived happiness in sociology and economics. This terminology is alternatively referred to as perceived social justice, perceived social fairness or social equity in most of the literature and it is an important index for social capital (Wegener, 1991; Sabbagh and Schmitt, 2016; Zhang et al., 2018; Liu and Jia, 2021).

2 This survey was conducted online in 2012 and only considered internet users. Most of the respondents are males.

3 Information set is information available from different sources and depends on which one forms perception as well as makes decisions.

## Appendix

**Table A1:** Variables description.

Variables	Definition	Measure
**Dependent Variable**		
*Respondent’s Subjective Social Justice (SSJ)*	How do you think about the current social justice in China?	Dummy: Agree or Not
		Five-Point Scale: From Strongly Disagree to Strongly Agree 1–5
**Key independent variable: Internet use**		
*Internet Utilization*	Whether use Internet or mobile Internet or not	Dummy: Agree or Not
*Internet Utilization Frequency*	How often the respondent uses Internet and mobile Internet during the past year?	Five-Point Scale: From Never use to use very often
**Control variables at the individual level**		
Male	Gender	Female = 0, Male = 1
Rural Hukou	Hukou Registration Status	Rural Hukou = 1, Urban Hukou = 0
Age	Age	Age in survey year
Han Ethnicity	Ethnicity: Whether is han or not	Han = 1, Minority = 0
Communism Party Member	Whether the respondent is Communist	Communist = 1, others = 0
Marriage Status	Marriage Status	If currently married with spouse, value is 1, otherwise 0
Health Status	Health status	Five-Point Scale: From very unhealthy to very healthy
Household Wealth	Whether Household Owns a House	If they own, value is 1; if not, value is 0
Sociability	How often the respondent goes out and socialize with friends	Five-Point Scale: From Never to very often
Atheist	Whether the respondent believes that God does not exist	Atheist = 1, others = 0
Log (Annual Income)	Logarithmic personal annual income in the past year	Logarithmic personal annual income
Comparative income	Individual household income comparing with local mean	Individual household income minus local average
Educational Attainment	Individuals’ Educational Completion	Five-Point Scale: From Lowest to highest educational level
Government Official	Whether the respondent works in public administration department	Government Official is 1, otherwise 0
Economic Social status	Self-evaluated household’s social status	Five-Point Scale: From Lower to Upper Class
**Control variables at the provincial level**		
Log (Real GDP per Capita)	Real gross provincial product per capita (base year 1978) Unit: 100 million yuan	Logarithmic real GDP per capita for each province at survey year
FDI Ratio	The ratio of foreign direct investment to gross domestic products at province level	FDI/GDP for each province at survey year
Log (population)	The total year-end provincial population Unit: 10 thousand	Logarithmic total year-end population for each province at survey year
Trade Volume Ratio	The percentage of trade volume out of GDP	the percentage of total trade volume out of GDP for each province at survey year
